# Low molecular weight polysialic acid binds to properdin and reduces the activity of the alternative complement pathway

**DOI:** 10.1038/s41598-022-09407-2

**Published:** 2022-04-06

**Authors:** Anahita Shahraz, Yuchen Lin, Joshua Mbroh, Jonas Winkler, Huan Liao, Marie Lackmann, Annemarie Bungartz, Peter F. Zipfel, Christine Skerka, Harald Neumann

**Affiliations:** 1 Neural Regeneration Unit, Institute of Reconstructive Neurobiology, Medical Faculty and University Hospital of Bonn, University of Bonn, Venusberg-Campus 1, 53127 Bonn, Germany; 2 Department of Infection Biology, Leibniz Institute for Natural Product Research and Infection Biology, Jena, Germany; 3 Infection Biology, Institute of Microbiology, Friedrich Schiller University, Jena, Germany

**Keywords:** Complement cascade, Immune evasion

## Abstract

Sialic acids as the terminal caps of the cellular glycocalyx play an essential role in self-recognition and were shown to modulate complement processes via interaction between α2,3-linked sialic acids and complement factor H. Previously, it was suggested that low molecular weight α2,8-linked polysialic acid (polySia avDP20) interferes with complement activation, but the exact molecular mechanism is still unclear. Here, we show that soluble polySia avDP20 (molecular weight of ~ 6 kDa) reduced the binding of serum-derived alternative pathway complement activator properdin to the cell surface of lesioned Hepa-1c1c7 and PC-12 neuroblastoma cells. Furthermore, polySia avDP20 added to human serum blocked the alternative complement pathway triggered by plate-bound lipopolysaccharides. Interestingly, no inhibitory effect was observed with monosialic acid or oligosialic acid with a chain length of DP3 and DP5. In addition, polySia avDP20 directly bound properdin, but not complement factor H. These data show that soluble polySia avDP20 binds properdin and reduces the alternative complement pathway activity. Results strengthen the previously described concept of self-recognition of sialylation as check-point control of complement activation in innate immunity.

## Introduction

The complement system is part of the innate immune system and plays a substantial role in maintaining immune-surveillance and tissue homeostasis^1,2^. The complement system is activated via three main pathways (1) classical, (2) lectin, and (3) alternative pathway^1^. The classical pathway is mediated by the binding of the complement component C1q to antigen–antibody immune complexes. The lectin pathway is activated by mannose-binding lectin recognition of glycol-motifs on the cell surface of damaged host and non-host cells; and the alternative complement pathway, which is constitutively active through the spontaneous cleavage of an internal C3-thioester bond in C3 via hydrolysis, will act as an amplifier on foreign or damaged surfaces^1–3^. Properdin is a positive regulator of the alternative pathway by stabilizing the C3-convertase (C3bBb) and a proposed pattern recognition molecule for activating the alternative pathway^3^. Moreover, properdin is produced by different subtypes of leukocytes and circulates in the blood as cyclic oligomers of monomeric subunits^3^.

Complement factor H acts as the negative regulator of the alternative complement pathway^4,5^. Factor H binds to α2,3-linked sialic acids that are present on healthy cells on the cellular glycocalyx^6^. Thereby, factor H binds to sialic acids and C3b on the cell surface at the same time, forming a C3b-factor H-sialic acid complex, which inhibits the C3-convertase formation via competition with factor B, disassembles C3-convertase via removing Bb, and accelerates degradation of cell surface C3b via factor I^4,5,7^.

Polysialic acid of mammals is a polymer of glycosidically linked α2,8-linked *N*-acetylneuraminic acid monomers with a degree of polymerization between 10 and approximately 200^8^. Polysialic acid is attached to several cellular glycoproteins (NCAM, neuropilin-2, etc.) on nervous and immune cells and contributes to synaptic plasticity and innate immune functions^8^. Furthermore, several bacteria can produce polysialic acid, including *Escherichia coli K1* (*E. coli K1*) and *Serogroup B Neisseria meningitidis*. Since for several decades it is known that the polysialic acid capsule of *E. coli K1* strain is responsible for preventing activation of the alternative complement pathway^9^ and the α2,8-linked polysialic acid capsule of *Serogroup B Neisseria meningitides* contributes to resistance against the bactericidal activity of normal human serum^10^. Therefore, the effect of polysialic acid on the complement cascade was studied^11^, but the mechanism of the complement-modulating effect of bacterial polysialic acid was not fully elucidated so far.

Oligosialic acid and polysialic acid with a α2,8-linkage are ligands for human sialic acid-binding immunoglobulin-like lectin 11 (SIGLEC11) and mouse sialic acid-binding immunoglobulin-like lectin-e (Siglec-e). Both, Siglecs are expressed on microglia^12–14^. Recently, we demonstrated that low molecular weight polysialic acid with an average degree of polymerization of 20 (polySia avDP20) had anti-inflammatory activity on SIGLEC11 expressing human mononuclear phagocytes and in an animal model of retinal laser lesion performed in humanized SIGLEC11-transgenic mice^15,16^. In addition, it was shown that polySia avDP20 also acts anti-inflammatory via Siglec-e on mouse microglia, but higher concentrations were needed for this effect compared to cultured human SIGLEC11-expressing phagocytes^16^. While most of the anti-inflammatory effects of polySia avDP20 were mediated via the SIGLEC11 receptor, we also observed a SIGLEC11‐independent modulation of the alternative complement pathway by polySia avDP20^16^.

Here, we studied the complement inhibitory effect of polySia avDP20 in more detail. We show that polySia avDP20 inactivates the property of properdin to bind to complement-susceptible murine hepatoma cells and rat neuroblastoma cells. Furthermore, we studied the interaction of polySia avDP20 with different components of the complement system and found a direct binding of polySia avDP20 to properdin. Moreover, we confirmed inhibition of the alternative complement pathway by polySia avDP20 with an inhibitory concentration IC50 of 5.8 µM. In addition, we found that this inhibitory effect was chain length dependent, since monosialic acid, as well as oligosialic acid of DP3 and DP5 had no effect.

## Materials and methods

### PolySia avDP20, biotin-conjugated polySia avDP20, high molecular weight polysialic acid, monosialic acid, oligosialic acid DP3 and DP5, low and high-sulfated dextran, and chondroitin sulfate

Polysialic acid (α2,8-linked *N*-acetylneuraminic acid) was obtained commercially (YC11298 colominic acid sodium salt, Carbosynth, UK) or collected from cultures of *Escherichia coli* B2032/82 serotype K1. Polysialic acid was purified as previously described^17^. High molecular weight polysialic acid was isolated by removing low molecular weight polysialic acid by filtration (Hydrosart, Sartorius Stedim Biotech GmbH). Low molecular weight polysialic acid with an average degree of polymerization 20 (polySia avDP20) was isolated via fast protein liquid chromatography (FPLC) from long-chain polysialic acid after heat-mediated hydrolysis as previously described^15^. Monosialic acid (Nacalai Tesque) and oligosialic acid DP3 and DP5 (Nacalai Tesque), high-sulfated dextran with molecular weight 5 kDa (TdB Labs), low-sulfated dextran with a molecular weight of 5 kDa (TdB Labs), and chondroitin sulfate with degree of polymerization 20 (iduron, UK) were commercially obtained.

The sialic acid concentration was determined via a thiobarbituric acid (TBA)-assay and chain length of polySia avDP20 was confirmed by gel electrophoresis and charged aerosol detector (CAD)-HPLC as previously described^15,18,19^. Endotoxin measurements were performed with Endosafe nexgen-PTS (Charles River) based on the manufacturer protocol. The obtained high molecular weight polysialic acid had an endotoxin contamination less than 100 EU/mg and the polySia avDP20 less than 50 EU/mg. Biotinylation of polySia avDP20 was performed as previously described^15^. Briefly, polySia avDP20 was oxidized to an aldehyde by sodium metaperiodate, and then hydrazide coupled biotin (Thermo Scientific) was conjugated to form a hydrazone bond. Purification of biotin-polySia avDP20 was carried out with a desalting column (HiTrap Desalting Column GE Healthcare).

### Size determination by gel electrophoresis

FPLC fractions of PolySia avDP20 were mixed 1:1 with 2 × Tris–Glycine loading buffer and loaded onto 4–20% Tris–Glycine gel (Bio-Rad). 12 µg highly sulphated dextran (10 kDa and 5 kDa, TdB Labs) served as molecular weight markers. A 100 bp DNA ladder (Thermo Fisher Scientific) served as an additional marker. Electrophoresis was performed on ice for 45 min at 120 V in 1 × Tris–Glycine buffer. Afterwards, the Tris–Glycine gel was recovered and placed in deionized water to wash out bromophenol blue band. Under protection from light, gel was stained with stains-all (Sigma Aldrich) solution in 50% methanol (Roth) in water (vv^−1^) until dextran markers were visible. Background was washed out with deionized water and gel was scanned with the default instrument settings for analysis (Lexmark CX727 scanner).

### Chain length determination by CAD-HPLC

Samples were diluted in deionized water and filtered with 0.22 µm pore size syringe filter (Corning). Samples were injected with a total volume of 500 µl and separated by a DNApac PA-100 column (Thermo Fisher Scientific) using an ÄKTA pure 25 L system (GE Healthcare). Deionized water (E_1_, Fresenius) and 4 M ammonium acetate (E_2_, Sigma Aldrich) were used as eluents at 1 ml min^−1^ flow rate. For the sample analysis the corona veo charged aerosol detector (CAD, Thermo Fisher Scientific) was used. The nebulizer gas was compressed nitrogen (≥ 99.8% purity, Linde Gas). The gas pressure was maintained at 60.7 psi. The gain range for the CAD was set to 100 pA. The evaporator temperature was set to 51 °C. The ion trap voltage was set to 20.1 V. The elution was performed by following gradient: t_0_ min = 0% (vv^−1^) E_2_; t_5_ min = 0% (vv^−1^) E_2_; t_15_ min = 10% (vv^−1^) E_2_; t_20_ min = 13% (vv^−1^) E_2_; t_35_ min = 17% (vv^−1^) E_2_; t_55_ min = 20% (vv^−1^) E_2_; t_100_ min = 26% (vv^−1^) E_2_; t_101_ min = 70% (vv^−1^) E_2_; t_115_ min = 70% (vv^−1^) E_2_; t_120_ min = 30% (vv^−1^) E_2_; t_120.1_ min = 0% (vv^−1^) E_2_; t_150_ min = 0% (vv^−1^) E_2_.

### Bioactivity test of polySia avDP20

For testing bioactivity of polySia avDP20, murine embryonic stem cell-derived microglia (ESdM) cells were seeded in 1.6 × 10^5^ density per well in a 12 well plate contain ESdM culture medium, which includes DMEM–F12 medium (Gibco), 0.48 mM l-glutamine (Gibco), 1% N_2_ supplement (GE Healthcare) and 1% penicillin/streptomycin (Gibco). After 24 h culturing, medium was changed and cells were treated with 1 µg/ml lipopolysaccharide (LPS) or LPS + 5 µM polySia avDP20 for 24 h. Total RNA was isolated from ESdM using RNeasy isolation kit (Qiagen). Then, SuperScript III reverse transcriptase (Thermo Fisher Scientific) and random primer mix (Roche Diagnostics) were employed for cDNA synthesis. Semi-quantitative RT-PCR was performed using 200 ng cDNA, SYBR GreenER qPCR SuperMix Universal (Thermo Fisher Scientific) and specific primers were added into a final reaction volume of 25 µl. The following primer pairs were used in real-time reactions:

- *Gapdh* F-AACTTTGGCATTGTGGAAGG, R-GGATGCAGGGATGATGTTCT;
- *Il1b* F-CTTCCTTGTGCAAGTGTCTG, R-CAGGTCATTCTCATCACTGTC;
- *Tnf* F-GACGTGGAACTGGCAGAAGA, R-ACTGATGAGAGGGAGGCCAT.

For PCR-amplifications, a Mastercycler ep Gradient S (Eppendorf) was used, and the results were evaluated by manufacturer’s software. Amplification specificity was confirmed by melting curve analysis, and the quantification of the obtained PCR products was performed by the ΔΔCt method with GAPDH as internal standard.

### Complement proteins and human serum

Human properdin was purchased from TECOmedical (Germany)/Complement Technology (USA). The complement factor C3, factor C3b, factor H, factor B, and properdin were purchased from Complement Technology (USA). Normal human serum (NHS) was obtained from healthy donors with informed consent or ordered from Complement Technology. All methods for use of human serum and complement factors were carried out in accordance with relevant national guidelines and regulations. All experimental protocols for use of human serum and complement factors were approved by the ethics committee of the University Hospital of Bonn.

### ELISA for detection of the interaction between polySia avDP20 and different complement factors

A high binding microtiter plate (Thermo Fisher Scientific) was used and 5 μg/ml of each purified complement proteins (properdin/Factor P, factor B, factor H, factor D, C3b, C3; C5, C6, C7, C8, C9, and C5b-9; Complement Technology) were immobilized on the plate. Then, 500 nM biotinylated polySia avDP20 was added. Binding of the biotinylated polySia avDP20 was detected by enzyme-conjugated streptavidin, followed by a color reaction. Similarly, properdin (5 µg/ml) was coated on the microtiter plate and incubated with increasing concentrations of polySia avDP20 (10–500 nM). Biotinylated polySia avDP20 was detected as described above. In addition, different concentrations (7.8–1000 nM) of properdin (factor P), factor H or bovine serum albumin (BSA) were coated on the ELISA plate and 500 nM biotinylated polySia avDP20 was added. Biotinylated polySia avDP20 was detected as described above. Binding of properdin and factor H to the ELISA plate was confirmed by specific monoclonal antibodies directed against factor P (Tecomedical) and factor H (clone T13) followed by secondary HRP-conjugated antibodies and the enzyme-mediated color reaction. All color reactions were measured by the ELISA reader (TECAN, Switzerland). All the values were normalized to the non-specific binding activity. All experiments were performed with triplicates for each sample and independently repeated three times.

### Rat pheochromocytoma cell line (PC-12)

PC-12 cells (ECACC 88022401, Sigma-Aldrich) were cultured as described by the manufacturer. The cell culture medium consisted of RPMI 1640 medium (Gibco), supplemented with 2 mM glutamine, 10% heat-inactivated horse serum, and 5% fetal bovine serum (all from Gibco). PC-12 cells grew as small clusters in suspension and were maintained in a density 5 × 10^5^ cells/ml in culture flasks coated by 0.1% collagen type IV (Sigma-Aldrich). For experiments, PC-12 cells were collected via 0.25% trypsin (Gibco) and seeded for culturing in collagen IV (Sigma-Aldrich) coated 4-chamber slides (Nunc Lab-Tek, Merck). After 24 h, cells were differentiated into neuron-like cells by addition of 50 ng/ml nerve growth factor (NGF; Sigma-Aldrich) diluted in 500 µl differentiating medium (RPMI 1640 medium contain 1% heat-inactivated horse serum) for 7 days before starting the experiment.

### Murine hepatoma cell line (Hepa-1c1c7)

Hepa-1c1c7 (Sigma-Aldrich) were cultured in alpha-minimum essential medium (MEM, Gibco) supplemented with 10% fetal bovine serum (FCS), 1% l-Glutamine and 1% Penicillin/Streptomycin (all from Gibco). For experiments, the adherent cell culture was kept till 80% confluency, then collected via 0.25% trypsin (Gibco), seeded into 4-chamber slides (Nunc Lab-Tek, Merck) in MEM containing limited serum levels (2% FCS) and then cultured for three further days to obtain Hepa-1c1c7 cells that are susceptible to human complement-mediated lysis.

### Analysis of polySia avDP20 effects on human properdin binding to lesioned cells

Hepa-1c1c7 and PC-12 cells were used to study the binding of human properdin on the lesioned cell surfaces. Susceptible cells were washed with 1 × phosphate buffered saline (PBS) and then incubated with pre-heated (1 h at 37 °C) 10% NHS (Complement Technology) for 5 min in gelatin veronal buffer with Mg^2+^ and Ca^2+^ (GVB ++; Complement Technology). Control cells were treated with pre-heated (1 h at 37 °C) 10% heat-inactivated NHS. PolySia avDP20 effect on human properdin binding was tested by co-incubation of the NHS with different concentrations of polySia avDP20 (1 h at 37 °C, 20 µM, 50 µM, 80 µM, 150 µM, 320 µM, 450 µM), then diluted tenfold in GVB++ lysis buffer to obtain the final concentration and added to cell culture for 5 min. This step was followed by a 1 × PBS washing step and fixation with 4% paraformaldehyde (PFA, Sigma-Aldrich). Properdin binding was detected and quantified by staining with an anti-human properdin antibody (mouse mAb 1340; described in Pauly et al.^20^), and a rabbit anti-neurofilament antibody (Sigma-Aldrich) as the primary antibodies for 2.5 h at RT followed by Alexa 647-conjugated goat anti-mouse antibody (1:500; Jackson ImmunoResearch) and Alexa 488-conjugated goat anti-rabbit (1:500; Invitrogen) for 1 h at RT. Cells were incubated with DAPI (1:10,000) and mounted with Moviol. The immunostained cells were visualized by confocal microscopy (Olympus) and quantified with the ImageJ software (NIH).

### Enzyme immunoassay for assessment of alternative complement pathway activity

The effects of polySia avDP20 on the alternative complement system was measured by a commercially available assay kit (#COMPL AP330 RUO) according to the manufacturers’ protocol. Briefly, NHS (provided by this kit or obtained from Complement Technology) was co-incubated with different concentrations of polySia avDP20 (1 h at 37 °C with 26 µM, 52 µM, 106 µM, 213 µM, 426 µM) or mono-/oligosialic acid/high molecular weight polysialic acid (1 h at 37 °C with 26 µM, 106 µM, 426 µM). Afterwards, the pre-incubated sera were diluted 13-fold in the diluent provided by the manufacturer to obtain the final concentration, and the whole mixtures were transferred to the pre-washed LPS-coated micro-titer plate (provided by the manufacturer) and incubated for 1 h at 37 °C. The wells were washed 3 times and then 100 µl of a specific alkaline phosphatase labelled antibody for terminal complement complex (TCC, C5b-9; provided by the manufacturer) was added and incubated for 30 min at RT. Afterwards, wells were washed 3 times, 100 µl of substrate solution was added for 30 min at RT and the optical density (OD) was measured at 405 nm by an ELISA reader (TECAN Infinite 200 PRO).

### Analysis of polySia avDP20 effects on human properdin binding to lesioned Hepa-1c1c7 cells via flow cytometry

Properdin (factor P), polySia avDP20 and different sera were preincubated for 30 min at 37 °C in a test tube, particularly the following samples were prepared: 10% NHS, 45 µM polySia, 2.5 µg/ml factor P, 2.5 µg/ml factor P + 45 µM polySia, 10% HI-NHS, 10% NHS + polySia (45 µM final concentration), 10% factor P- depleted serum, 10% factor P-depleted serum + polySia (45 µM final concentration), 10% factor P-depleted serum + 2.5 µg/ml factor P, 10% factor P-depleted serum + 2.5 µg/ml factor P + 45 µM polySia). Compstatin (45 µM, Selleckchem), a small molecule inhibitor of complement factor C3, was used as control. The different samples were then added to the susceptible Hepa-1c1c7 cells for 5 min. Cells were blocked for 1 h in blocking solution containing 10% bovine serum albumin and 5% normal goat serum. Cells with the different samples were then divided into two tubes and either incubated for 1 h with mouse anti human factor P antibody (1:1000, Bio-Rad) or the respective control antibody. Afterwards, all samples were incubated for 30 min on ice and in darkness with Alexa647-conjugated goat anti mouse IgG (1:500, Jackson ImmunoResearch) in blocking buffer. Samples were measured with BD Accuri C6 Plus and analysed using FlowJo v10 software.

### Statistical analysis

Data are shown as boxplot of at least n = 3 from independent experiments. Data were analyzed by Levene's test for variance equality, then followed by one-way ANOVA subsequently Bonferroni post hoc test using IBM SPSS Statistics 23.0 software. Results are considered significant as *p < 0.05, **p < 0.01, or ***p < 0.001.

## Results

### Production and purification of low molecular weight polysialic acid (polySia avDP20)

**Figure 1:**
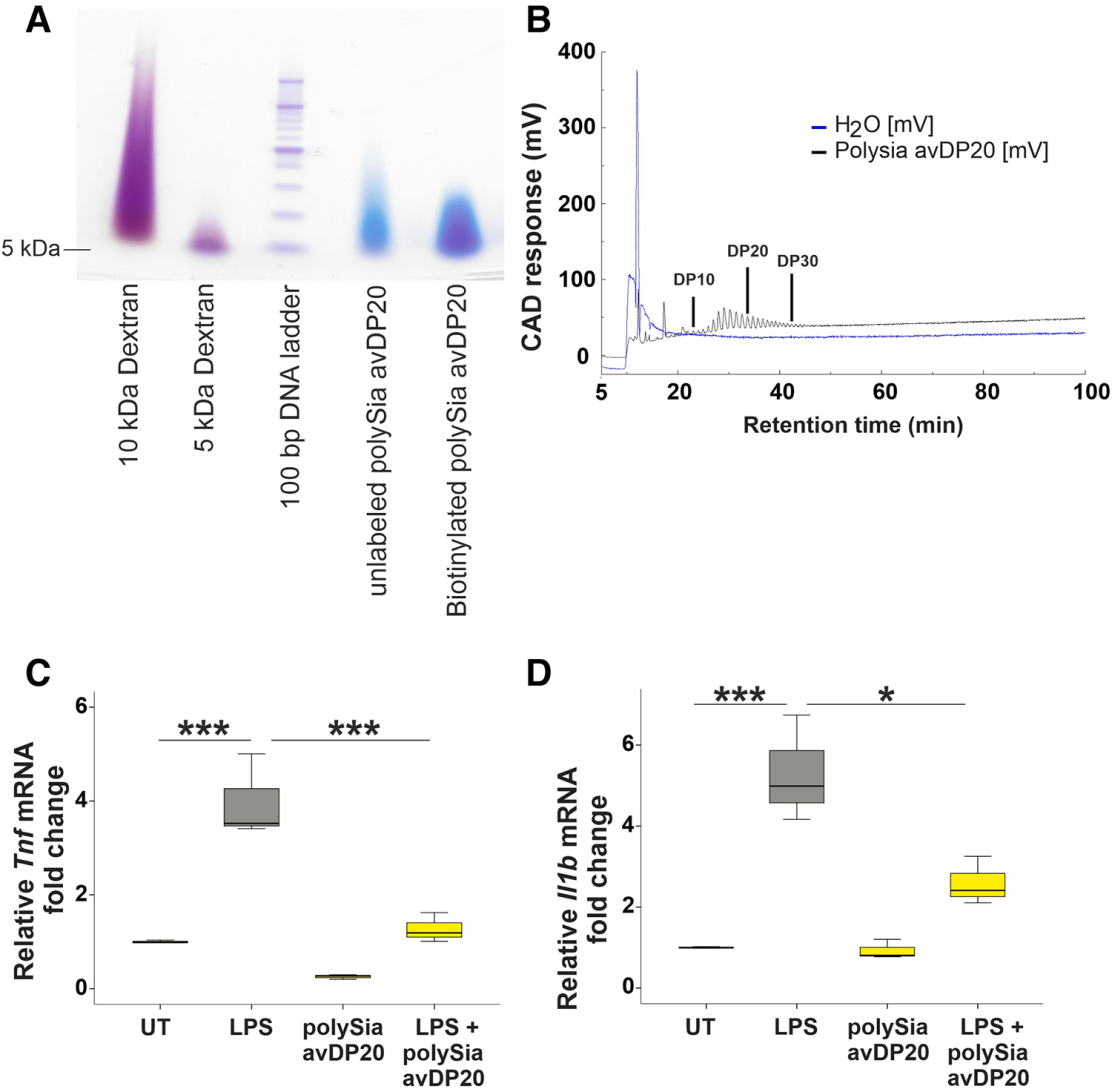
Purification and bioactivity of polySia avDP20. (**A**) Tris–Glycine gel electrophoresis to determine the size of the polysialic acid fraction, that was obtained by heat-mediated hydrolysis of bacterial polysialic acid, tangential flow filtration and anion exchange chromatography. (**B**) Analysis of polySia avDP20 by charged aerosol detector (CAD) high-performance liquid chromatography. (**C**) Anti-inflammatory bioactivity of polySia avDP20 was determined by measurement of the pro-inflammatory cytokine gene transcription. LPS-induced *Tnf* transcript level was attenuated by application of polySia avDP20. (**D**) Elevated transcript level of *Il1b* was also attenuated by application of polySia avDP20. Data are shown as boxplot (min./Q1/median/Q3/max.) of n = 3 independent experiments normalized to the untreated control. One-way ANOVA followed by Bonferroni post hoc test; *p < 0.05, ***p < 0.001. *UT* untreated, *DP* degree of polymerization, *LPS* lipopolysaccharide.

Polysialic acid was either purchased or obtained from the supernatant of the *E. coli* strain serotype K1 cultured in a bioreactor. Therefore, bacteria were separated by microfiltration and the polysialic acid containing supernatant was collected and purified^15,19^. Afterwards, controlled fragmentation of polysialic acid was performed by mild heat mediated hydrolysis and the fragments with different chain lengths were isolated by a gradient fast protein liquid chromatography (FPLC) with a Q-membrane adsorber^15,19^. The size of the obtained polySia was determined by molecular weight in the gel electrophoresis (Fig. 1A), and as chain length distribution in a gradient high pressure liquid chromatography (HPLC) equipped with a charged aerosol detector (CAD) (Fig. 1B). In addition, anti-inflammatory bioactivity of polysialic acid with an average degree of polymerization 20 (polySia avDP20) was confirmed by using murine embryonic stem cell derived microglial cells (ESdM), as previously described^15,16^. Data showed that polySia avDP20 (5 µM) reduced the lipopolysaccharide (LPS)-stimulated gene transcription of *tumor necrosis factor* (*Tnf*) and *interleukin-1b* (*Il1b*). In detail, the application of LPS increased the *Tnf* transcripts from 1 ± 0.02 to 3.98 ± 0.5 (mean ± SEM; p < 0.001; Fig. 1C), while polySia avDP20 co-treatment reduced it to 1.3 ± 0.18 (mean ± SEM; p = 0.001; Fig. 1C). In addition, application of LPS increased the *Il1b* transcripts from 1 ± 0.01 to 5.3 ± 0.7 (mean ± SEM; p = 0.001; Fig. 1D) and polySia avDP20 co-treatment reduced it to 2.6 ± 0.34 (mean ± SEM; p = 0.012; Fig. 1D).

Thus, polySia avDP20 obtained and purified from bacteria was confirmed to act anti-inflammatory in a Siglec-e expressing cell culture bioassay.

### PolySia avDP20 prevented binding of properdin to lesioned Hepa-1c1c7 and PC-12 cell surfaces

**Figure 2:**
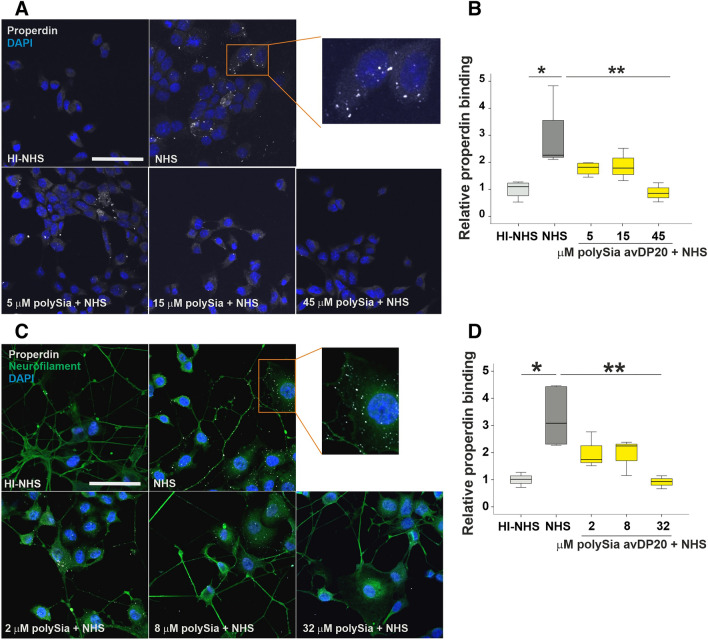
PolySia avDP20 reduced binding of human properdin to lesioned cell surfaces. (**A**) Immunohistochemistry staining of properdin binding to the cell surfaces of lesioned Hepa-1c1c7 cells (properdin is visualized in gray/white). Cells were treated with 10% human serum (NHS). Representative images are shown from n > 3 independent experiments. (**B**) Quantification of properdin binding based on intensity measurements. PolySia avDP20 was able to reduce the binding of properdin to the lesioned cells in a concentration dependent manner. (**C**) Immunohistochemistry staining of properdin binding to the cell surfaces of lesioned PC-12 cells (properdin is visualized in gray/white). Cells were treated with 10% human serum (NHS). Representative images are shown from n > 3 independent experiments. (**D**) Quantification of properdin binding based on intensity measurements. PolySia avDP20 was able to reduce the binding of properdin to the lesioned cells in a concentration dependent manner. Scale bar: 50 µm. Data are shown as boxplot (min./Q1/median/Q3/max.) of n = 3 independent experiments, and are normalized to the heat inactivated normal human serum. One-way ANOVA followed by Bonferroni post hoc test; *p < 0.05, **p < 0.01. *HI-NHS* heat inactivated normal human serum, *NHS* normal human serum, *PolySia* polySia avDP20.

To investigate the effects of polySia avDP20 on the alternative complement system, we used a murine hepatoma cell line (Hepa-1c1c7) and a rat pheochromocytoma cell line (PC-12) that are susceptible to human complement-mediated lysis by serum deprivation. Serum deprivation can lead to non-uniform reduction of basal metabolic activity and energy failure^21,22^. Thus, serum starvation over time triggers a diverse spectrum of cell membrane damage that subsequently will lead to localized opsonization of complement factors including properdin. Both cell lines were treated by gelatin veronal buffer with Mg^2+^ and Ca^2+^ (GVB++ buffer) and incubated with 10% normal human serum (NHS) that contains properdin. The binding of properdin to the lesioned cells surface was visualized by immunostaining with a specific antibody and confocal microscopy (Fig. 2). Binding of properdin to the lesioned cells surface upon incubation in NHS was detected for both cell types by the specific antibody directed against properdin (Fig. 2A,C). Next, we analyzed whether polySia avDP20 might interfere with the binding of human properdin to the lesioned cells surface. Therefore, polySia avDP20 was added to NHS in different concentrations and pre-incubated for 1 h. The mixtures were then added to the cells for 5 min, again using the GVB++ buffer. PolySia avDP20 prevented the binding of properdin to the lesioned cells surface of both cell types, in a concentration dependent manner. In the Hepa-1c1c7 cell line, the binding of human properdin was reduced from 2.9 ± 0.6 to 1.8 ± 0.1 (mean ± SEM) by a final concentration of 5 µM polySia avDP20, to 1.8 ± 0.2 (mean ± SEM) by 15 µM polySia avDP20, and to 0.9 ± 0.1 (mean ± SEM; p = 0.007) by 45 µM polySia avDP20 (Fig. 2B). In the PC-12 cell line, binding of human properdin to lesioned cells was reduced from 3.3 ± 0.48 to 2 ± 0.38 (mean ± SEM) by a final concentration of 2 µM polySia avDP20, to 1.9 ± 0.38 (mean ± SEM) by 8 µM polySia avDP20, and to 0.9 ± 0.1 (mean ± SEM; p = 0.009) by 32 µM polySia avDP20 (Fig. 2D). In both cell lines, heat inactivated serum (HI-NHS) used as control did not show any effect.

To further investigate properdin binding, we performed flow cytometry experiments with lesioned Hepa-1c1c7 cells incubated with purified properdin (factor P), NHS or factor P-depleted serum (Supplementary Fig. S1). Flow cytometry analysis showed binding of purified properdin to the lesioned Hepa-1c1c7 cells, that was inhibited after coincubation of properdin with polySia avDP20 (Supplementary Fig. S1A). In detail, polySia avDP20 reduced the binding of properdin to the lesioned cells from 78.4 ± 0.92% (mean ± SEM; factor P) to 7.1 ± 1.92% (mean ± SEM; polySia avDP20 + factor P). In addition, polySia avDP20 reduced physiological properdin binding present in NHS to lesioned cells (from 11.9 ± 1.9% to 2.6 ± 0.5%; mean ± SEM; Supplementary Fig. S1B). In addition, supplementation of properdin to factor P-depleted serum increased the properdin binding to the cell surface of the lesioned Hepa-1c1c7 cells, which was reverted after pre-incubation of properdin with polySia avDP20 (Supplementary Fig. S1C). In detail polySia avDP20 reduced the binding of properdin to the lesioned cells from 65.2 ± 3.7% (mean ± SEM; factor P-depleted serum + factor P) to 35 ± 12% (mean ± SEM; polySia avDP20 + factor P-depleted serum + factor P). Data show that addition of polySia avDP20 to human serum reduces binding of properdin to lesioned cells surface of human complement-susceptible Hepa-1c1c7 and PC-12 cells.

### PolySia avDP20 inhibited alternative complement activation

**Figure 3:**
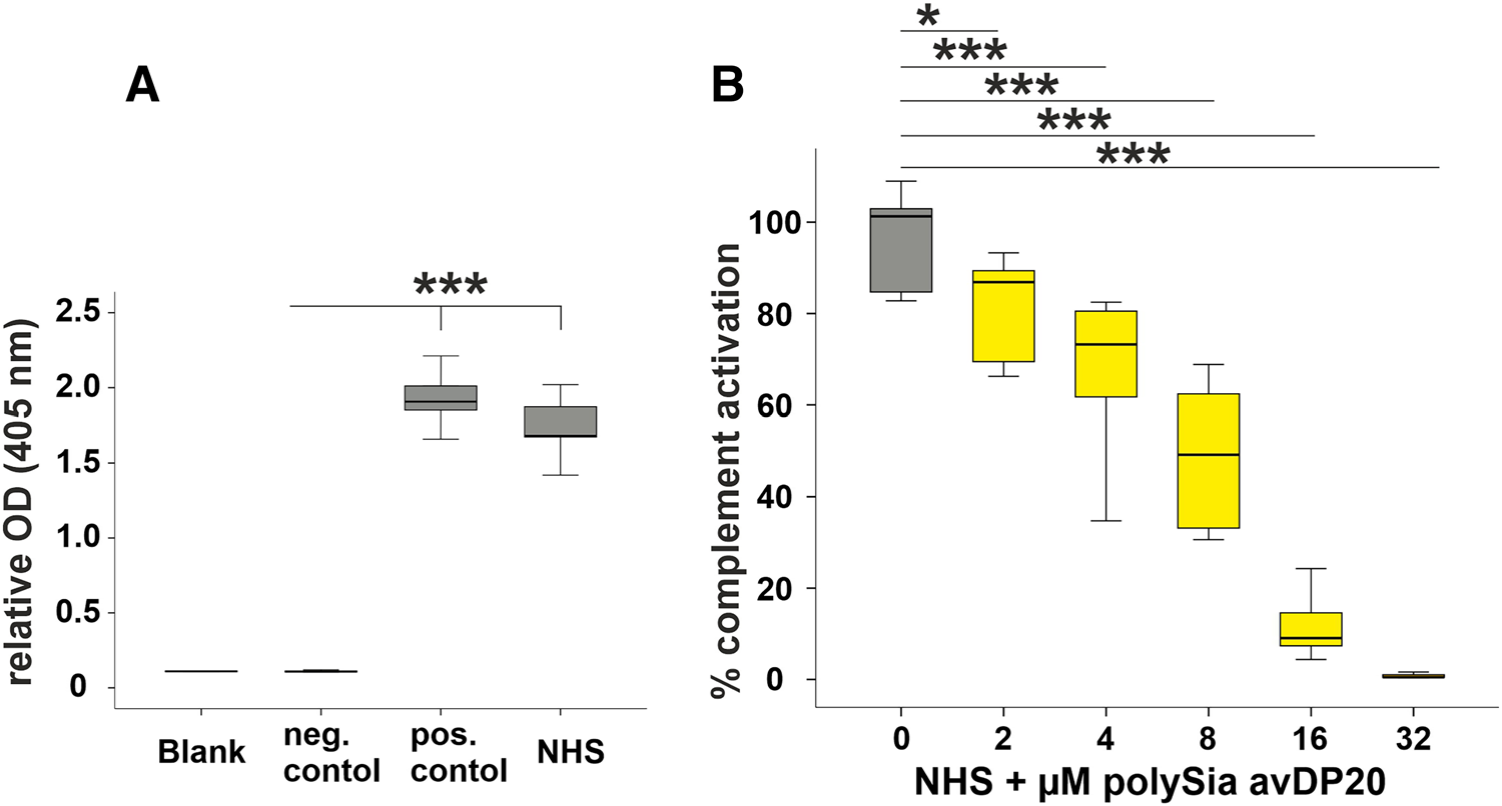
Soluble polySia avDP20 reduced the activity of the NHS (10%) to form the TCC (C5b-9), as determined by ELISA. (**A**) NHS was capable to induce C5b-9 formation similar to a positive control. (**B**) PolySia avDP20 was co-incubated with NHS and then activation of the alternative pathway was measured by detection of C5b-9 formation using the ELISA. PolySia avDP20 reduced the activity of the TCC formation in a dose-dependent manner. Data are shown as boxplot (min./Q1/median/Q3/max.) of n = 5 independent experiments, and are normalized to the untreated blank control. One-way ANOVA followed by Bonferroni post hoc test; *p < 0.05, ***p < 0.001. *NHS* normal human serum.

To further investigate the inhibitory effect of polySia avDP20 on alternative complement pathway (AP) activity, we measured the inhibition of this pathway via a commercially available complement system kit. This kit is based on the formation and accumulation of the terminal complement complex (TCC, C5b-9) subunits using a LPS-coated ELISA plate format. The applied NHS promoted TCC formation (1.7 ± 0.1; mean ± SEM; p < 0.001) similarly to the positive control (1.9 ± 0.12; mean ± SEM; p < 0.001) supplied by the manufacturer (Fig. 3A). Furthermore, a negative control confirmed that the TCC was specifically activated (0.1 ± 0.004; mean ± SEM; Fig. 3A). Factor P-depleted serum was used as an additional control and was unable to activate the AP (Supplementary Fig. S2). Next, NHS was co-incubated with different concentrations of polySia avDP20 for 1 h. Afterwards, this pre-treated serum was added to the ELISA plate to initiate the activation of the AP. Results revealed that polySia avDP20 reduced the AP activation in all concentrations used. In detail, the percentage of AP activation in NHS was reduced in from 96.1 ± 5.2% to 81 ± 5.5% (with a final concentration of 2 µM polySia avDP20; mean ± SEM; p = 0.023), to 66.5 ± 8.7% (with a final concentration of 4 µM polySia avDP20; mean ± SEM; p < 0.001), to 49 ± 7.6% (with a final concentration of 8 µM polySia avDP20; mean ± SEM; p < 0.001), to 11.9 ± 3.5% (with a final concentration of 16 µM polySia avDP20; mean ± SEM; p < 0.001), and to 0.8 ± 0.2% (with a final concentration of 32 µM polySia avDP20; mean ± SEM; p < 0.001) (Fig. 3B). The inhibitory concentration IC50 of polySia avDP20 was calculated based on a logarithmic equation as 5.8 µM.

Thus, data show that addition of soluble polySia avDP20 to serum inhibits the terminal complement complex formation in a concentration dependent manner.

### Monosialic acid and oligosialic acids (DP3, DP5) failed to inhibit alternative complement pathway

**Figure 4:**
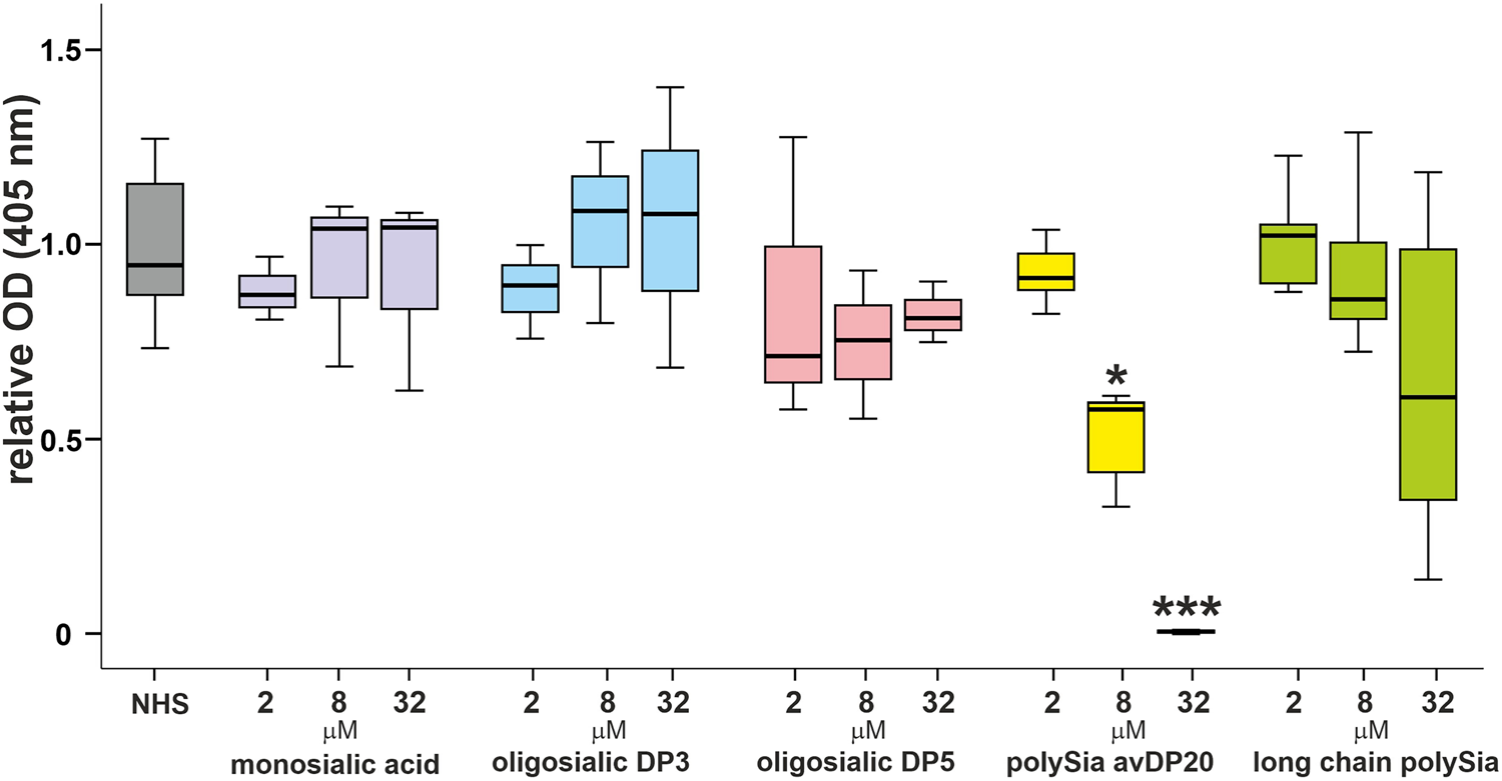
Monosialic acid, oligosialic acid, soluble polySia avDP20, and soluble long chain polySia was co-incubated with 10% NHS. Afterwards, activation of the alternative pathway was measured by detection of TCC formation using the ELISA. PolySia avDP20 reduced TCC formation in a dose-dependent manner. Data are shown as boxplot (min./Q1/median/Q3/max.) of n ≥ 3 independent experiments, and are normalized to normal human serum. One-way ANOVA followed by Bonferroni post hoc test; *p < 0.05, ***p < 0.001. *NHS* normal human serum.

To analyze whether the inhibitory effect of polysialic acid is chain length dependent, we also determined the AP activity by measuring TCC formation in the presence of monosialic acid, oligosialic acid (DP3, DP5) and high molecular weight polysialic acid. Again, NHS was co-incubated for 1 h with different concentrations of monosialic acid, oligosialic acids or polysialic acid having different chain length. Afterwards, these pre-treated sera were added to the LPS-coated ELISA plate to initiate the AP. Results showed that monosialic acid as well as oligosialic acid (DP3, DP5) had no inhibitory effect, while high molecular weight polySia had a low inhibitory activity on TCC formation. PolySia avDP20 used as positive control regularly showed a clear reduction of TCC formation. In detail, the monosialic acid treatment regardless of the concentration used did not significantly change the TCC formation (1 ± 0.07, mean ± SEM in NHS treated group; 0.9 ± 0.05, 2 µM monosialic acid; 0.94 ± 0.13, 8 µM monosialic acid; 0.91 ± 0.15, 32 µM monosialic acid). A similar behavior was observed with oligosialic acids. The oligosialic acid DP3 did not affect TCC formation (1 ± 0.07; mean ± SEM in NHS group; to 0.9 ± 0.07, 2 µM oligosialic acid DP3; 1.05 ± 0.14, 8 µM oligosialic acid DP3; 1.05 ± 0.2, 32 µM oligosialic acid DP3). Also, oligosialic acid DP5 did not affect TCC formation (0.8 ± 0.21, 2 µM oligosialic acid DP5; 0.7 ± 0.1, 8 µM oligosialic acid DP5; 0.8 ± 0.05, 32 µM oligosialic acid DP5). However, polySia avDP20 reduced TCC formation from 1 ± 0.07 to 0.5 ± 0.1 (8 µM polySia avDP20; mean ± SEM; p = 0.014), and 0.01 ± 0.006 (32 µM polySia avDP20; mean ± SEM; p < 0.001). The high molecular weight polySia only showed a slight trend for inhibiting the TCC with a relative high variability (Fig. 4).

As additional control, we performed experiments with other negatively charged polysaccharides. High-sulfated dextran with molecular weight 5 kDa (2 µM, 8 µM, 32 µM), low-sulfated dextran with a molecular weight of 5 kDa (2 µM, 8 µM, 32 µM) and chondroitin sulfate with degree of polymerization 20 (2 µM, 8 µM, 32 µM) were tested in comparison to polySia avDP20 (2 µM, 8 µM, 32 µM) in the AP assay kit (Supplementary Fig. S3). Only high-sulfated dextran and polySia avDP20 inhibited the TCC formation, thus indicating that a high negative charge might be one required property for this observed bioactivity.

Thus, no complement inhibitory effect of monosialic acid, oligosialic acid DP3 and DP5, low-sulfated dextran (5 kDa) and chondroitin sulfate DP20 was observed, while polySia avDP20 and high-sulfated dextran inhibited the TCC formation.

### PolySia avDP20 interacts with human properdin, but not with factor H

**Figure 5:**
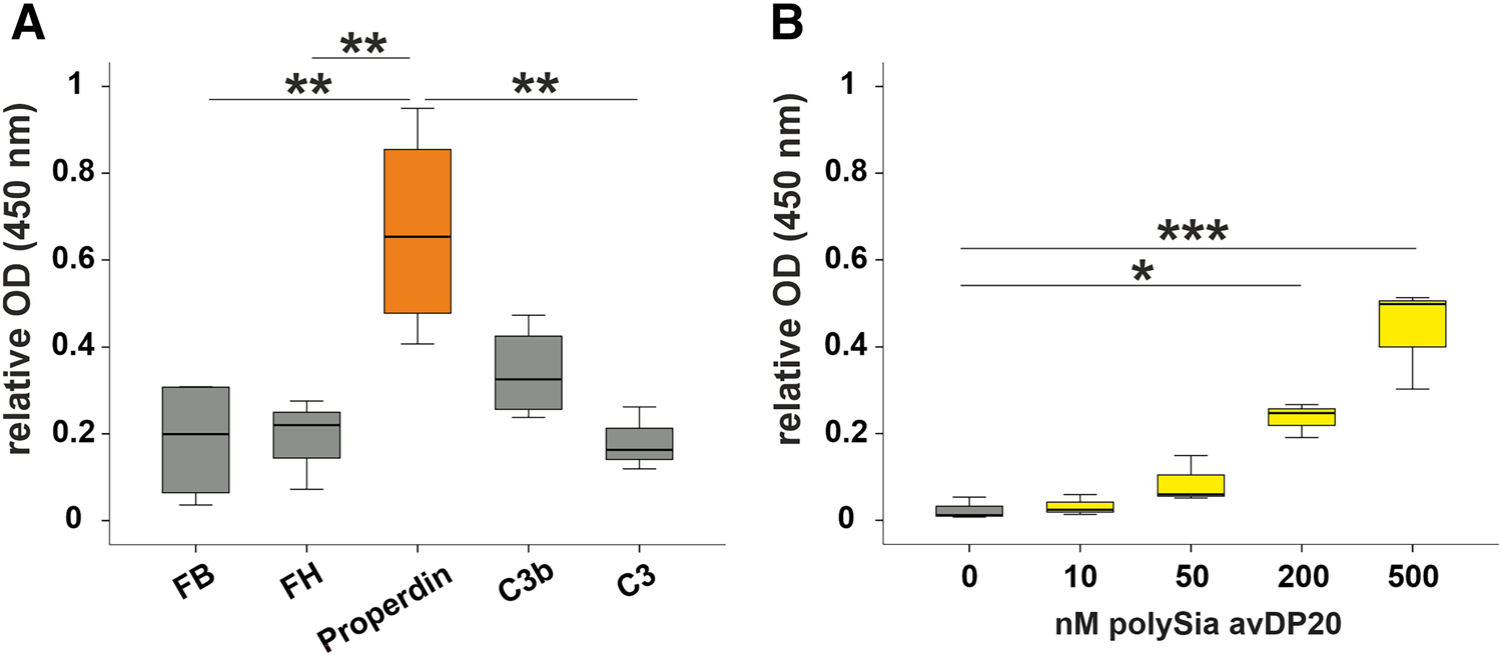
Interaction between polySia avDP20 and human properdin. (**A**) Factor B (FB), factor H (FH), properdin, active complement component C3b, and complement factor 3 (C3) were captured on a microtiter ELISA plate. Biotinylated polySia avDP20 showed binding to properdin, while no binding to factor B, factor H and C3 was observed. (**B**) Properdin (5 μg/ml; 0.09 μM) was bound on a microtiter plate and then incubated with different concentrations of biotinylated polySia avDP20. Binding of polySia avDP20 to properdin was determined by ELISA. Data are shown as boxplot (min./Q1/median/Q3/max.) and are normalized to the mean of the negative controls. One-way ANOVA followed by Bonferroni post hoc test; *p < 0.05, **p < 0.01, ***p < 0.001.

As a next step, we studied the potential interaction partners of polySia avDP20 for inhibiting complement and TCC formation. Binding activity of biotin-conjugated polySia avDP20 to complement components factor B, factor H, properdin, C3b and C3, that were bound to an ELISA plate, was analyzed. Therefore, polySia avDP20 was conjugated with biotin and the molecular weight was confirmed by gel electrophoresis (Fig. 1A). The biotinylated polySia avDP20 (500 nM) captured to the distinct complement factors was visualized by a streptavidin-conjugated color reaction in an ELISA (Fig. 5A). Results showed that biotinylated polySia avDP20 interacted with immobilized properdin (0.66 ± 0.11; mean ± SEM; Fig. 5A), while no binding to factor H (0.2 ± 0.04; mean ± SEM; p = 0.003), factor B (0.19 ± 0.07; mean ± SEM; p = 0.002), and C3 (0.18 ± 0.03; mean ± SEM; p = 0.002) was observed. A slight, but not significant, binding of biotinylated polySia avDP20 to C3b was also visible (Fig. 5A).

In addition, an assay was performed to investigate the interaction between polySia avDP20 and other terminal complement components (Supplementary Fig. S4). Compared to binding of polySia avDP20 to properdin (1 ± 0.04; mean ± SEM), polySia avDP20 did not interact with factor D (0.03 ± 0.02; mean ± SEM; p < 0.001) or the terminal complement components C5 (0.01 ± 0.02; mean ± SEM; p < 0.001), C6 (0.04 ± 0.02; mean ± SEM; p < 0.001), C8 (− 0.01 ± 0.02; mean ± SEM; p < 0.001) and C9 (0.05 ± 0.01; mean ± SEM; p < 0.001). A slight binding of PolySia avDP20 to complement component C7 (0.3 ± 0.06; mean ± SEM; p < 0.001) and the C7 containing complex C5b-9 (0.23 ± 0.02; mean ± SEM; p < 0.001) was observed (Supplementary Fig. S4).

**Figure 6:**
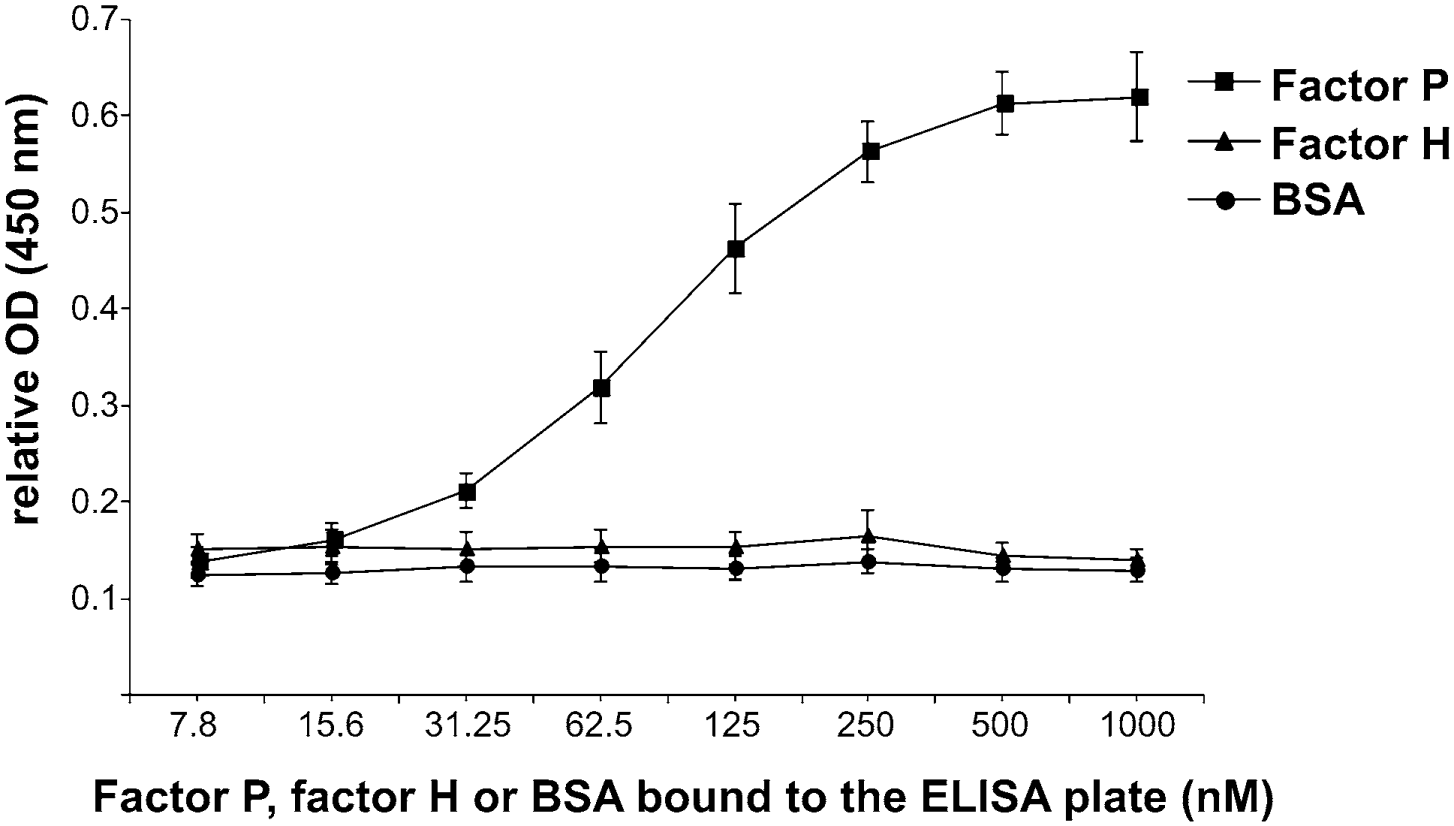
Concentration dependent binding of polySia avDP20 to properdin, but not factor H. An ELISA plate was coated with different concentrations from 7.8 to 1000 nM of factor P (properdin), factor H or bovine serum albumin (BSA). Biotinylated polySia avDP20 (500 nM) was added. PolySia avDP20 bound to factor P (coating protein concentration of 31.25 nM or higher), but not to factor H or the BSA control. Data are shown as mean ± SEM of n = 3 independent experiments.

Next, we added different concentrations of biotinylated polySia avDP20 to 5 µg/ml purified properdin that was bound to the ELISA plate (Fig. 5B). The biotinylated polySia avDP20 bound to properdin in a concentration dependent manner. In detail, the respective OD values were increased from 0.024 ± 0.01 (in negative control; mean ± SEM) to 0.23 ± 0.02 (with 200 nM biotin-polySia avDP20; mean ± SEM; p = 0.02), and to 0.44 ± 0.067 (with 500 nM biotin-polySia avDP20; mean ± SEM; p < 0.001). To further investigate whether polySia avDP20 could bind to physiological forms of properdin in serum, different concentrations of NHS were added to a 5 μg/ml polySia avDP20-coated ELISA plate (Supplementary Fig. S5). We observed that a minimum concentration of 10% NHS was needed to see the binding of properdin to polySia avDP20 (Supplementary Fig. S5). Finally, we tested different concentrations (7.8–1000 nM) of properdin, factor H or bovine serum albumin (BSA) coated to the ELISA plate and determined the binding of 500 nM biotinylated polySia avDP20 (Supplementary Fig. S4). Binding of polySia avDP20 to properdin was concentration-dependent with an almost linear range between 25 to 250 nM of properdin coated to the ELISA plate. No binding of polySia avDP20 to factor H or BSA was observed over the whole concentration range tested (Fig. 6).

In summary, biotinylated polySia avDP20 showed selective binding to properdin, but no interaction with factor H. In addition, binding of polySia avDP20 to properdin was concentration dependent. The binding strength of properdin to polySia avDP20 was relatively high, although this could be related to a multimerization process commonly seen in interactions between carbohydrates and proteins.

## Discussion

In this study, we observed that soluble polySia avDP20 can act as a regulator of the alternative complement pathway (AP). Preincubation of human serum with polySia avDP20 prevented the binding of human serum properdin on the surface of susceptible murine hepatoma cells and rat neuroblastoma cells and inhibited AP in an ELISA-based in vitro assay. In addition, polySia avDP20 showed direct binding to properdin. Furthermore, we observed that the inhibitory activity of polySia on the AP was dependent on the degree of polymerization of polysialic acid. No effect was observed by monosialic acid, nor by oligosialic acid with chain lengths DP3 and DP5.

Sialic acids, including polysialic acid, as terminal cap on the cellular glycocalyx have an important role in homeostasis of immune cells, development of synapses, and neural development. Sialic acids are known to be recognized by sialic acid-binding immunoglobulin-like lectins (Siglec) receptors that are present on several immune cells and mainly act inhibitory on the cells. Particularly, polysialic acid has been shown to act anti-inflammatory via human SIGLEC11 and mouse Siglec-e on mononuclear phagocytes^14,15^. In line with these previous studies, here we confirmed the anti-inflammatory function of polySia avDP20 on mouse stem cell derived microglial cells. In addition, we identified a second ligand for polySia avDP20, namely properdin. Properdin is also part of the innate immune system and acts as a positive regulator of the AP by stabilizing the C3-convertase (C3bBb) and as proposed pattern recognition molecule for activating the AP^3^. Recently, the role of sialylation in modulating the complement system in different organs has been shown^23^. In fetal-maternal interface, presence of sialic acids will regulate maternal complement homeostasis and protect the embryo against developmental defects caused by maternal AP activation^23^. In an in vivo study, glucosamine-2-epimerase/N-acetylmannosamine kinase (GNE, an essential enzyme for sialic acid biosynthesis) heterozygous mice with less sialylation showed reduced synapse numbers and less microglial cell arborization, effects that were dependent on complement factor C3^24^. In addition, enzymatic removal of sialic acids from the neural glycocalyx in vitro led to C1q opsonization and clearance of neurites via microglial cells^25^. Here, we observed that polySia avDP20, which was added to human serum was able to inhibit binding of the serum factor properdin on the surface of susceptible cells. Data also showed that purified properdin alone was capable to bind to lesioned cells, and polySia avDP20 can neutralize this activity of properdin, but we cannot exclude that purified properdin is pre-activated or that additional complement components promote the binding of the purified properdin to the lesioned cells^3^.

Properdin has been proposed to act as pattern recognition molecule, to stabilize the C3-convertase and amplify the AP, that could lead to formation of a terminal complement complex (TCC, C5b-9)^3^. Here, we showed that polySia avDP20 prevented TCC formation in a concentration dependent manner with an inhibitory concentration of IC50 = 5.8 µM, which was dependent on properdin. Binding of carbohydrates to proteins are often regarded as low affinity interactions. Here, polySia avDP20 appears to be exceptional. Interestingly, polysialic acid is highly anionic (expected pKa ~ 4.8^26^), while properdin is highly cationic (isoelectric point > 9.5^27^), suggesting a tendency of multimerization and giving a plausible explanation for this binding between properdin and polySia avDP20. Interestingly, high-sulfated dextran was also able to inhibit AP activity, while low-sulfated dextran had no effect. Nevertheless, we don’t understand the exact molecular events how polySia avDP20 inhibits the AP. Preincubation of polySia avDP20 with the NHS for at least 15 min was essential to observe the inhibitory activity^16^.

A previous study suggested that *N*-acetylneuraminic acid (Neu5Ac or NANA) as monosialic acid might have an inhibitory effect on the activation of C3 and the following complement cascade. However, relative high unphysiological concentrations of *N*-acetylneuraminic acid (between 12.5 and 100 mM) were required to inhibit C3 or factor B (FB) breakdown, while the levels of factor H (FH) and factor (FI) were not markedly changed^28^. In our studies an approximately 1000 × times lower concentration of polySia avDP20 was bioactive in blocking the alternative complement cascade (IC50 = 5.8 µM). In addition, monosialic acid, as well as oligosialic acids (DP3, DP5), low-sulfated dextran (5 kDa), and chondroitin sulfate DP 20 did not show any inhibitory function in the concentration range (2–32 µM) studied here. However, we cannot exclude that monosialic acid or oligosialic acid might also show inhibitory activity on the AP at a higher concentration level. Overall, our data indicate that polySia avDP20 and to a lower extent high molecular weight polysialic acid were capable to inhibit the AP in a low micromolar concentration range.

Presence of glycoconjugates on cell surfaces is essential for proper function of factor H as one of the major negative regulator of the complement pathway^2,4,5^. Cell membrane associated sialylated glycoconjugates increase the affinity of factor H to bind C3b, and via this function prevent further assembly of complement components^4^. Factor H does not bind to α2,8-linked sialic acids, but recognizes α2,3-linked sialylated oligosaccharides^6^. This is in line with our current data, in which we show no binding of polySia avDP20 to factor H. Thus, there might be a redundant and multivariate regulation of the complement system by different forms of sialylated glycoconjugates, oligosaccharides, and polysialic acid interacting either with factor H or properdin. Properdin (the potent activator of AP, that leads to C3b binding to factor B) and factor H (the main regulator of AP, that leads to C3b cleavage by factor I) are the main opposing players of the AP, and both of these factor show binding to factor C3b^29,30^. Properdin also shows direct competition with factor I by having overlap in their binding site to C3b^31^. In addition, properdin binding to C3b not only leads to a conformational change, which displaces the binding site of regulatory protein like factor I and factor H, but also stabilizes proconvertases and indirectly makes it harder to degrade C3b from factor B^31,32^.

Recently, several studies described shedding of polysialic acid (polySia) from its protein core such as neural cell adhesion molecule (NCAM) and possible functions of soluble polySia in the local microenvironment^33^. Soluble polySia can bind to diverse growth factors like brain derived neurotrophic factor (BDNF), vascular endothelium growth factor (VEGF) and basic fibroblast growth factor (bFGF), and thereby help to protect them from proteolysis, concentrate them in form of a reservoir, or modulate their interaction with their respective receptors^34,35^. In addition, α2,8-linked polySia can act as a cellular receptor for human adenovirus 52, showing the highest binding strength with DP ≥ 3^36^. Furthermore, polySia with different chain lengths (approximately DP20–DP40) is present in human serum and might act anti-inflammatory via reducing cytotoxicity related to histones, which could be released upon neutrophil extracellular trap formation^37^. As described before, soluble polySia avDP20 binds to SIGLEC11 receptor and modulates microglial cells function^15^. Furthermore, shedding of polysialylated proteins including polysialylated e-selectin-ligand1 and polysialylated neurophilin2 shedded from microglia upon inflammatory stimulation can in turn modulate innate immune responses^38^. We now identified another role for soluble polySia avDP20 as a modulator of the complement pathway by direct binding to properdin.

Based on our current study, we now propose a new mechanism of action for polysialic acid. Soluble polySia avDP20 inhibits AP by binding to properdin and thereby reduces the activity of the AP, as well as the capacity of complement to opsonize the surface of lesioned cells.

## Supplementary Information

Supplementary Figures.

## Data Availability

The data that support the findings of this study are available from the corresponding author upon reasonable request.
