# Supplementary material for: Low molecular weight polysialic acid binds to properdin and reduces the activity of the alternative complement pathway
Source: Sci Rep. 2022 Apr 6;12:5818. doi: 10.1038/s41598-022-09407-2 (PMC8987038; doi:10.1038/s41598-022-09407-2)

Supplementary material

Low molecular weight polysialic acid binds to properdin and reduces the activity of the alternative complement pathway

Anahita Shahraz [1, *], Yuchen Lin [2, *], Joshua Mbroh [1], Jonas Winkler [1], Huan Liao [1], Marie Lackmann [1], Annemarie Bungartz [1], Peter F. Zipfel [2,3], Christine Skerka [2, *], and Harald Neumann [1, *]

[1] Institute of Reconstructive Neurobiology, Medical Faculty and University Hospital of Bonn, University of Bonn, Bonn, Germany

[2] Department of Infection Biology, Leibniz Institute for Natural Product Research and Infection Biology, Jena, Germany

[3] Infection Biology, Institute of Microbiology, Friedrich Schiller University, Jena, Germany

* both authors contributed equally

# Correspondence to:

Harald Neumann, Neural Regeneration Unit, Institute of Reconstructive Neurobiology, Medical Faculty and University Hospital of Bonn, University of Bonn, Venusberg-Campus 1, 53127 Bonn, Germany, email: harald.neumann@uni-bonn.de, Tel. 49.228.6885.541

Supplementary figures

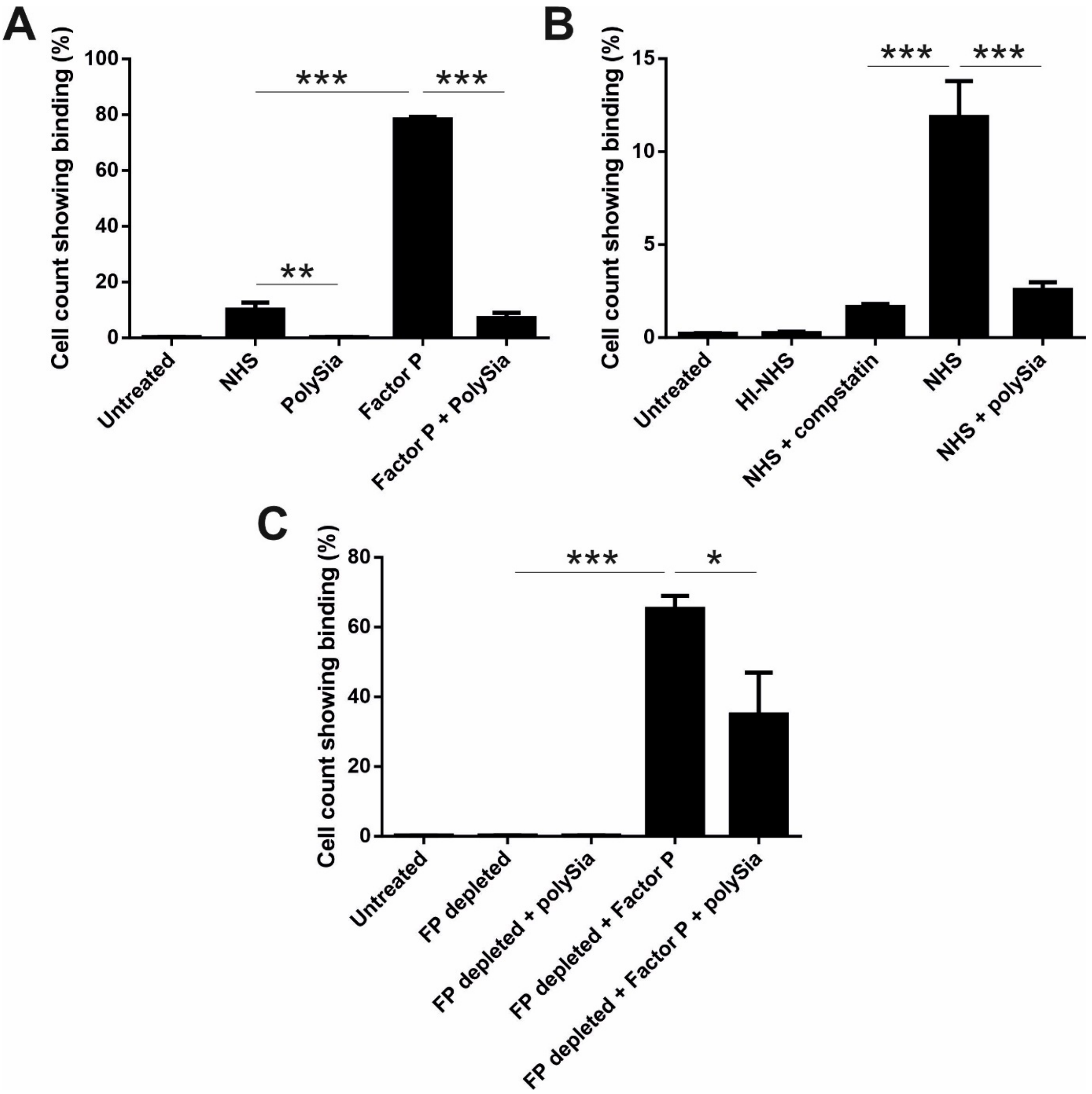

Supplementary figure S1. Low molecular weight polysialic inhibited binding of properdin to lesioned cells.

A. Factor P (2.5 µg/ml) was added to lesioned cells (Hepa-1c1c7) and binding of factor P to the cell surface was analyzed by flow cytometry with an antibody directed against factor P. Preincubation of factor P with 45 µM polySia avDP20 (polySia) reduced the relative percentage of cells with binding of properdin on the cell surface from 78.4 % ± 0.9 % to 7.1 % ± 1.9 % (mean ± SEM).

B. The lesioned Hepa-1c1c7 cell line treated with normal human serum (NHS) showed binding of factor P to a minority of cells (11.9 % ± 1.9 %; mean ± SEM),

which was reduced by addition of 45 µM polySia avDP20 (polySia; 2.6% ± 0.5 %; mean ± SEM).

C. Addition of Factor P (FP; 2.5 µg/ml) to factor P-depleted serum revealed an increased properdin binding to lesioned cell surfaces (65.2 % ± 3.7 %; mean ± SEM), that was reduced after addition of 45 µM polySia avDP20 (polySia) (35 % ± 12 %; mean ± SEM).

Data are shown as mean ± SEM of n=3 independent experiments. One-way ANOVA followed by Bonferroni *post hoc* test; *p < 0.05, **p < 0.01, ***p < 0.001. NHS: normal human serum; HI-NHS: heat-inactivated normal human serum; factor P: properdin; polySia: polySia avDP20.

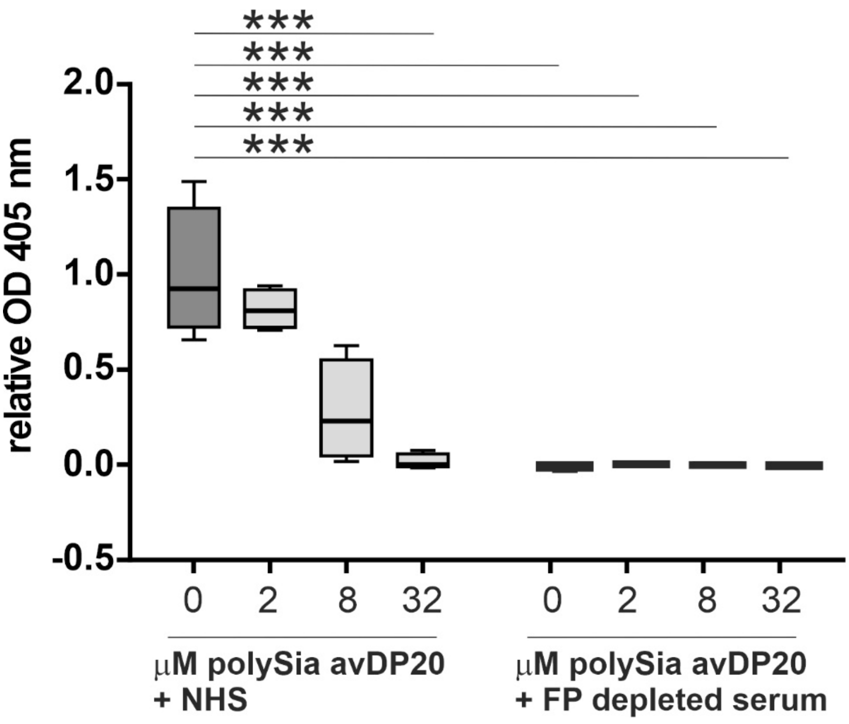

Supplementary figure S2. Factor properdin-depleted serum was unable to activate the alternative complement pathway.

Factor properdin (FP)-depleted serum (Complement Technology, US) with and without polySia avDP20 was tested in the alternative complement pathway assay (AP; Wieslab assay kit). PolySia avDP20 reduced the AP activity of NHS, while FP-depleted serum was unable to activate the AP, independent on the addition of polySia avDP20. Data are shown as boxplot (min./ Q1/ median/ Q3 /max.) of n=4 independent experiments normalized to the normal human serum. One-way ANOVA followed by Bonferroni *post hoc* test; ***p < 0.001. NHS: normal human serum. FP: factor properdin.

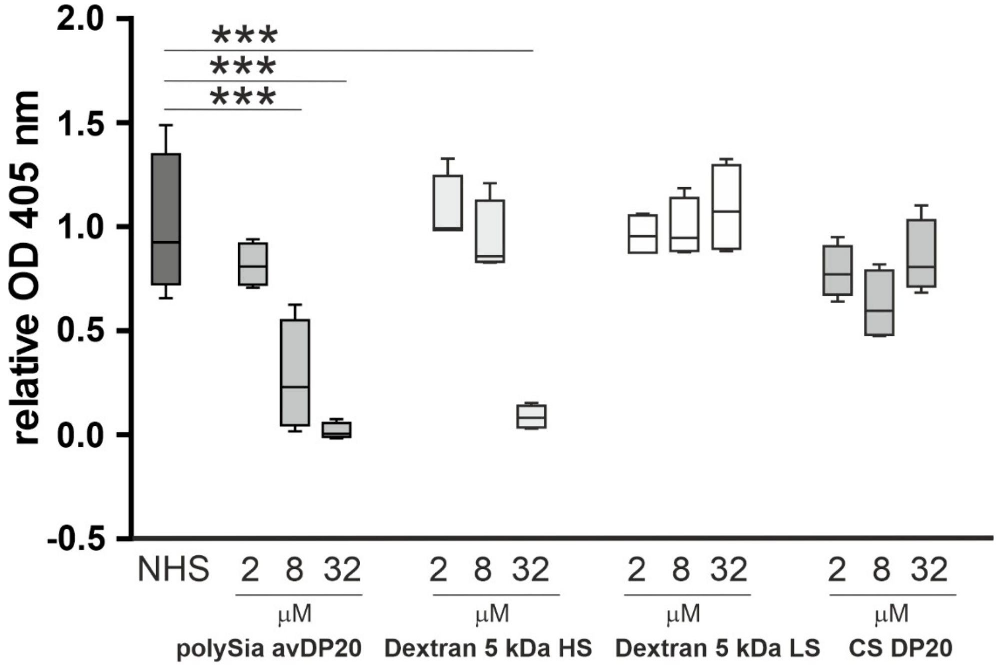

Supplementary figure S3. Effects of different polysaccharides on terminal complement complex formation.

High-sulfated dextran with molecular weight of 5 kDa (Dextran 5 kDa HS; TdB Labs), low-sulfated dextran with a molecular weight of 5 kDa (Dextran 5 kDa LS; TdB Labs) and chondroitin sulfate with degree of polymerization 20 (CS DP20; iduron, UK) were tested in comparison to polySia avDP20 using the alternative complement pathway assay (Wieslab assay kit). Results show that high-sulfated dextran and polySia avDP20 inhibited terminal complement complex (TCC) formation, thus indicating that the high negative charge might be one required property for this observed bioactivity. Data are shown as boxplot (min./Q1/median/Q3/max.) of n=4 independent experiments normalized to the normal human serum. One-way ANOVA followed by Bonferroni *post hoc* test; ***p < 0.001. NHS: normal human serum.

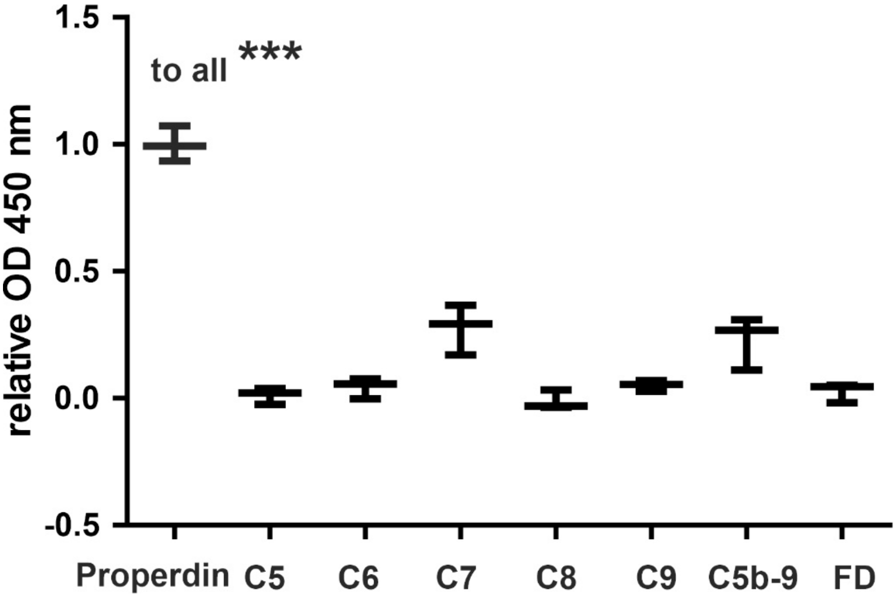

Supplementary figure S4. Interaction between polySia avDP20 and properdin in comparison to other terminal complement factor components.

Biotinylated polySia avDP20 was added to an ELISA plate that was coated with distinct complement factor components. PolySia avDP20 showed the highest binding to properdin. No binding of polySia avDP20 to complement 5 (C5), C6, C8, C9 and complement factor D was detected. A slight binding of polySia avDP20 to C7 and C5b-9 complex was observed. Data are shown as boxplot (min./ Q1/ median/ Q3/ max.) of n=3 independent experiments normalized to properdin binding. One-way ANOVA followed by Bonferroni post hoc test; ***$p < 0.001$. FD: complement factor D.

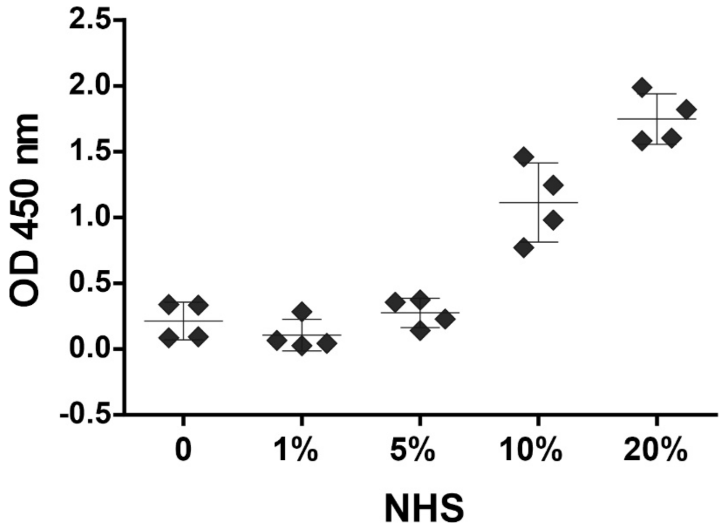

Supplementary figure S5. Interaction between polySia avDP20 and normal human serum.

Different concentrations of normal human serum (NHS) were added to an ELISA plate that was pre-coated with 5 µg/ml polySia avDP20. Binding of properdin to the polySia avDP20 coated plate was determined by a properdin-specific antibody. A minimum concentration of 10% NHS was needed to detect a pull-down of physiological properdin from the serum to the polySia avDP20 coated plate. Data are shown as mean +/- SD of n=4 data points from two independent experiments.

Original images to figure 1a (with decreasing exposure times)

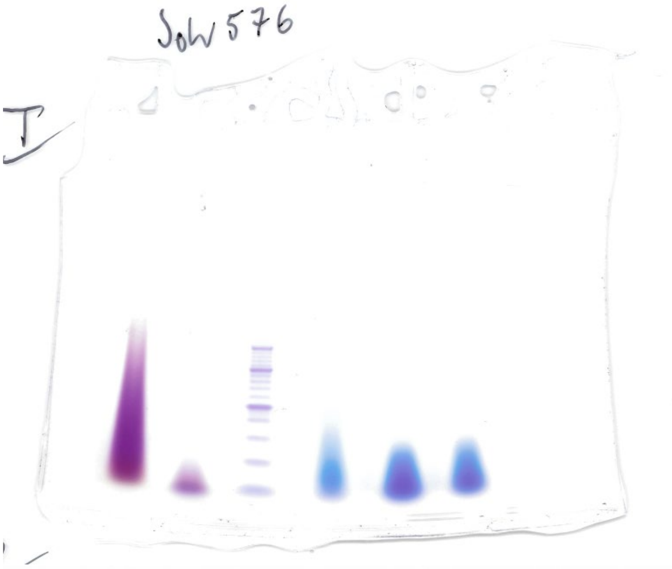

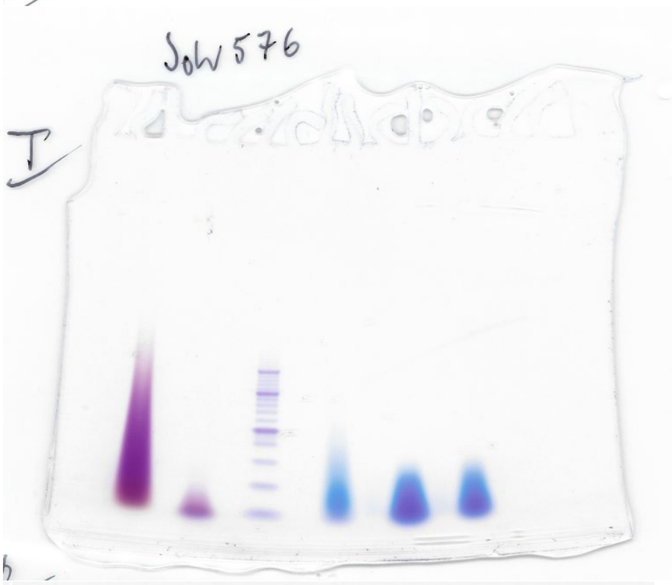

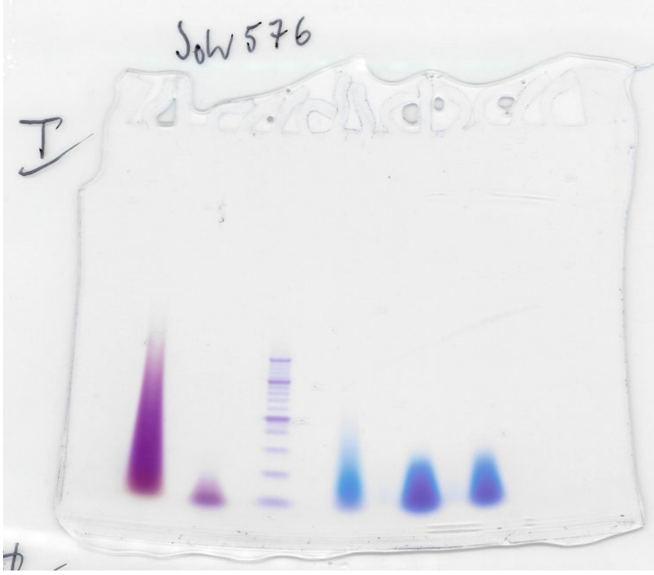

Original images to figure 1b

JoW296 H2O

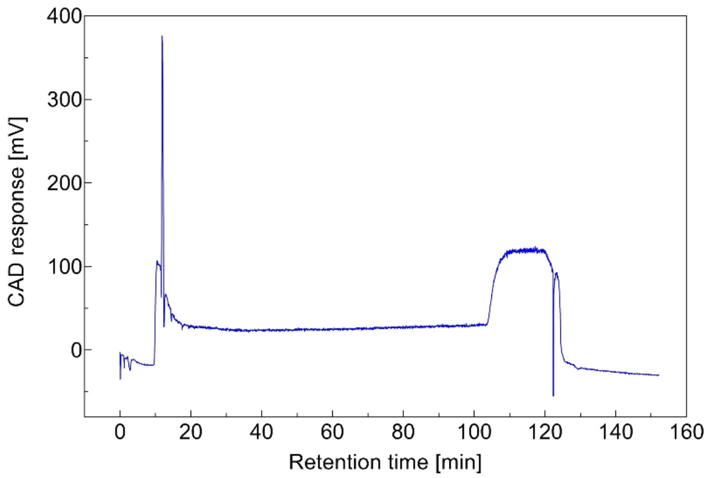

JoW296 - Batch #29f

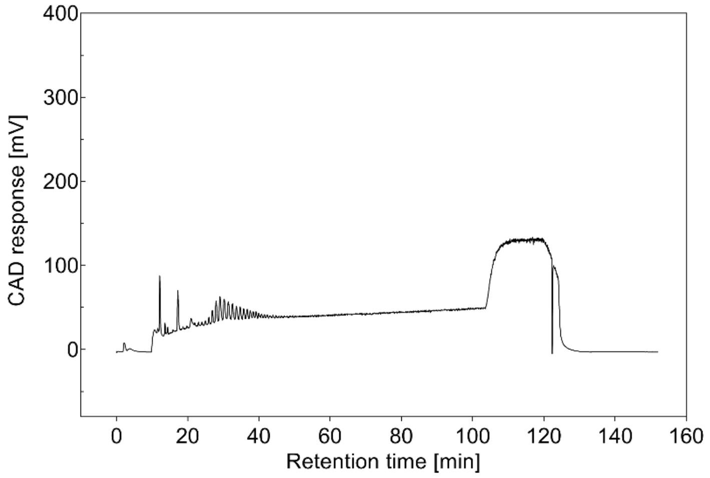

Supplement: Supplementary file 1 — Supplementary Figures. [file 41598_2022_9407_MOESM1_ESM.pdf]
